# Organo-mineral associations in chert of the 3.5 Ga Mount Ada Basalt raise questions about the origin of organic matter in Paleoarchean hydrothermally influenced sediments

**DOI:** 10.1038/s41598-019-53272-5

**Published:** 2019-11-13

**Authors:** Julien Alleon, David T. Flannery, Nicola Ferralis, Kenneth H. Williford, Yong Zhang, Jan A. Schuessler, Roger E. Summons

**Affiliations:** 1 Department of Earth, Atmospheric and Planetary Sciences, Massachusetts Institute of Technology, Cambridge, Massachusetts USA; 2 Jet Propulsion Laboratory, California Institute of Technology, Pasadena, California USA; 3 Department of Materials Science and Engineering, Massachusetts Institute of Technology, Cambridge, Massachusetts USA; 4 GFZ German Research Centre for Geosciences, Potsdam, Germany; 5 Present Address: Now at Institute of Earth Sciences, University of Lausanne, Lausanne, Switzerland

**Keywords:** Palaeontology, Carbon cycle

## Abstract

Hydrothermal and metamorphic processes could have abiotically produced organo-mineral associations displaying morphological and isotopic characteristics similar to those of fossilized microorganisms in ancient rocks, thereby leaving false-positive evidence for early life in the geological record. Recent studies revealed that geologically-induced alteration processes do not always completely obliterate all molecular information about the original organic precursors of ancient microfossils. Here, we report the molecular, geochemical, and mineralogical composition of organo-mineral associations in a chert sample from the ca. 3.47 billion-year-old (Ga) Mount Ada Basalt, in the Pilbara Craton, Western Australia. Our observations indicate that the molecular characteristics of carbonaceous matter are consistent with hydrothermally altered biological organics, although significantly distinct from that of organic microfossils discovered in a chert sample from the ca. 3.43 Ga Strelley Pool Formation in the same area. Alternatively, the presence of native metal alloys in the chert, previously believed to be unstable in such hydrothermally influenced environments, indicates strongly reducing conditions that were favorable for the abiotic formation of organic matter. Drawing definitive conclusions about the origin of most Paleoarchean organo-mineral associations therefore requires further characterization of a range of natural samples together with experimental simulations to constrain the molecular composition and geological fate of hydrothermally-generated condensed organics.

## Introduction

Some of the most commonly accepted lines of evidence for early life on Earth are exposed in the Pilbara Craton, Western Australia. There, stromatolites of the 3.43 Ga Strelley Pool Formation display diverse morphotypes in a non-varying paleoenvironment, and consistency of morphotypes across varying paleoenvironments^1–3^. In addition, they contain organic matter having carbon and sulfur isotopic compositions consistent with biological sources exploiting various metabolisms^4–6^. Finally, the associated sedimentary cherts host organic microstructures interpreted as microfossils based on their morphological, elemental, isotopic and molecular characteristics^7–13^. Altogether, within the framework of documented sedimentary and metamorphic histories, these observations support microbial activity in shallow-water environments on the early Earth by 3.43 Ga.

On many occasions, however, identifying the source of carbonaceous matter found in Archean rocks remains challenging, and commonly results in conflicting interpretations^14,15^, as illustrated by the long-standing debate regarding the origin of carbonaceous microstructures from the 3.46 Ga Apex chert. There, filamentous and spherical microstructures were interpreted as either remnants of Earth’s oldest microorganisms^16–19^, or as abiotic carbon-mineral associations formed during hydrothermal circulation of fluids^20–22^.

In parallel, experimental studies have demonstrated that hydrothermal processes can generate organo-mineral microstructures with size and shapes similar to those attributed to microorganisms^23,24^, and can abiotically reduce inorganic carbon into organic molecules with ^13^C-depleted isotopic compositions similar to those expected for biological processes^25–27^. Consequently, and although carbonaceous matter is widely reported from hydrothermal and sedimentary cherts in the Paleoarchean formations of the Pilbara Craton, both biotic and abiotic scenarios have been proposed for its origin^20,28–32^.

Thermally-induced structural and molecular transformations of organic biomolecules associated with diagenesis and metamorphism generally lead to intense alteration of molecular biosignatures^33–35^. Yet, diagenesis and metamorphism do not always completely obliterate the initial molecular characteristics of organic fossils. Molecular heterogeneities attributed to distinct organic precursors indeed persist despite burial temperature and pressure conditions generally considered to be incompatible with such preservation^13,36–38^. Significantly, hydrogen-, oxygen- and nitrogen-rich functional groups have been preserved in organic microfossils from chert of the Strelley Pool Formation, despite experiencing metamorphic conditions typical of prehnite-pumpellyite to lower greenschist facies, which has been assumed to be incompatible with such molecular preservation^13^. Experimental studies demonstrated that the mineralogy associated with microorganisms during their fossilization plays a critical role in their morphological, chemical and molecular transformations upon simulated diagenesis^39–46^. In this instance, entombment of microorganisms within amorphous opaline silica was experimentally shown to significantly limit their molecular degradation when they are subsequently exposed to thermal conditions typical of prehnite-pumpellyite to lower greenschist facies metamorphism in the range 250–300 °C^44,46^.

The vast majority of Paleoarchean cherts in the Pilbara Craton were exposed to these metamorphic conditions, and thus have the potential for preserving molecular information relevant to assessing the source of organic matter. Bulk scale molecular techniques, such as py-GC-MS and NMR, have been used to evaluate the origin of organic matter by identifying molecular features attributable to paleobiological activity^32,47^. Yet the application of bulk analytical techniques to ancient organic materials can be problematic because of contamination introduced during post depositional geological processes, exposure in outcrop, archival and/or during the preparation of samples^48–51^.

For the present work, we used advanced spatially-resolved spectroscopy/spectrometry techniques to characterize *in situ* the chemistry of organo-mineral associations discovered in a sample of black chert collected from the ca. 3.47 Ga Antarctic Creek Member (ACM), which lies within the mafic-ultramafic volcano-sedimentary succession of the Mount Ada Basalt, Warrawoona Group, Pilbara Craton, Western Australia^52–54^.

**Figure 1:**
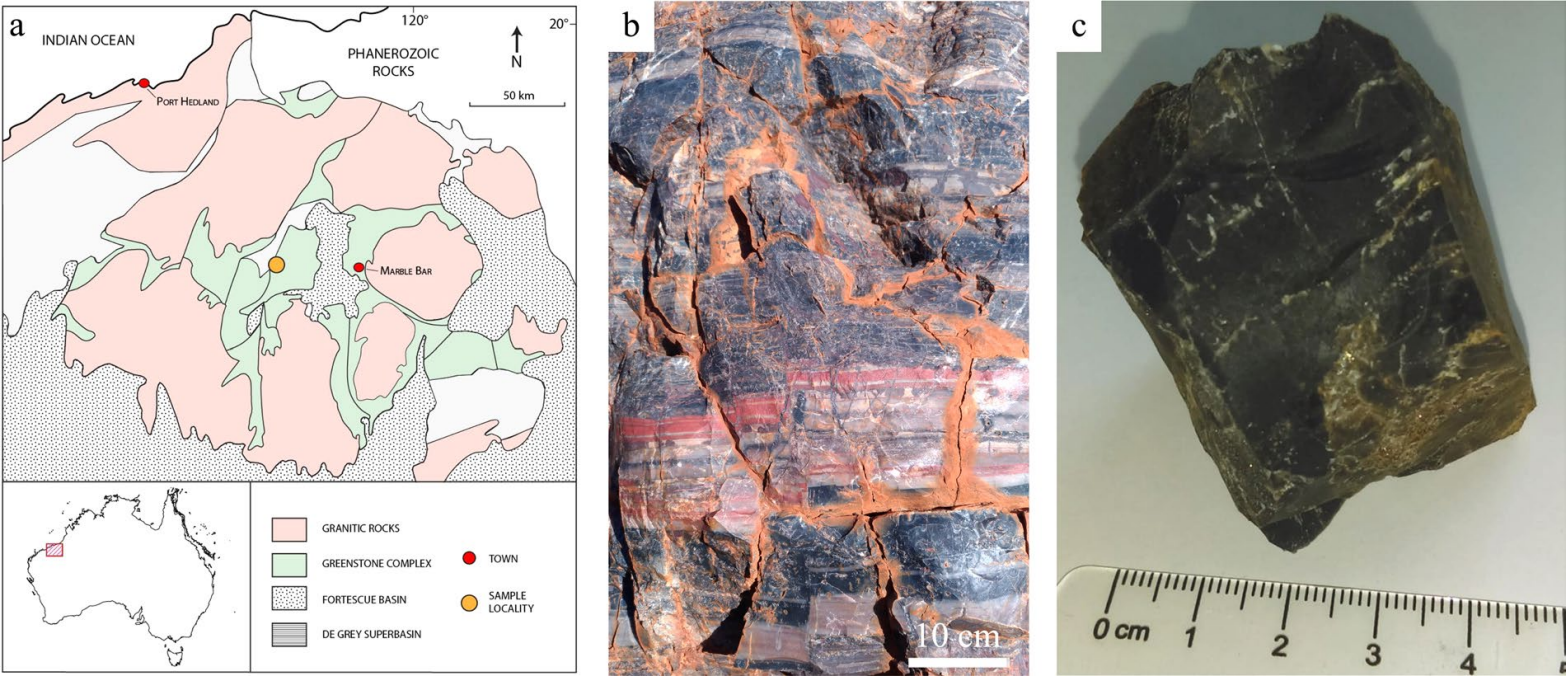
Context map (**a**); photograph of the outcrop (**b**) where the Ada chert studied here was sampled (**c**).

The ACM is one of several thin sedimentary horizons interbedded within up to 2.5 km of pillowed metabasalt and dolerite/gabbro sills of the Mt. Ada Basalt. Volcanism and associated hydrothermalism may have been related to the uplift of the Muccan Granitic Complex^55^. At our field site, which is located approximately 4 km north of Antarctic Creek in the North Pole Dome (Fig. 1), the ACM is present as a 5–20 m thick unit consisting of chert, jaspillite, lapilli tuff, carbonate, arenite, conglomerate and impact spherule layers. A ^207^Pb/^206^Pb date of 3470 ± 1.9 Ma is reported by Byerly, *et al*.^56^, who isolated zircons that are present within the spherule-bearing chert of the ACM approximately 17 km ENE of our field site. Spherules reported from the ACM are thought to have originated in a significant bolide impact event^57,58^, which resulted in a tsunami with an amplitude sufficient to generate a breccia in a deep sea environment where pillow basalt and sedimentary chert were accumulating below wave base. Petrographically similar spherules are reported from a similarly aged (3.47 Ga) unit within the Hoogenoeg Formation, Barberton Greenstone Belt, South Africa^56,59^.

## Results

### Petrography and geochemistry

**Figure 2:**
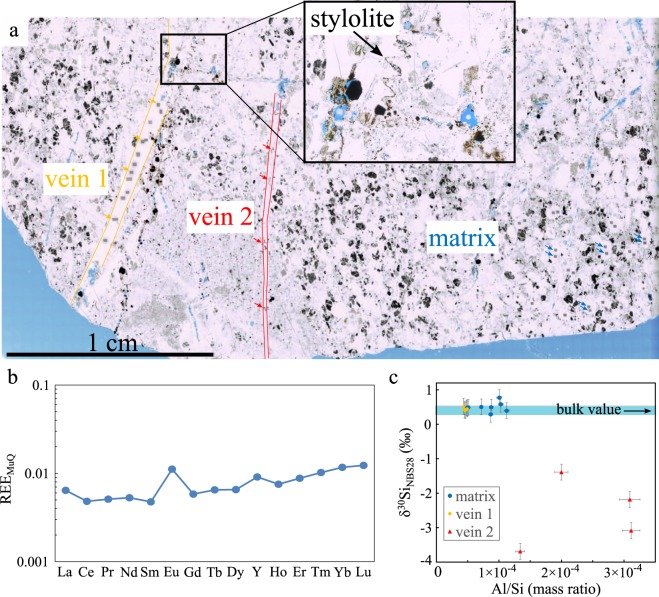
Petrography and Geochemical analyses. (**a**) Photomicrograph of the petrographic thin section of the studied Mount Ada Basalt chert sample, showing the relations between the chert matrix and the cross-cutting silica veins. Note the presence of stylolites, suggesting a sedimentary origin of the precursor of the chert matrix before silicification. The blue stain visible in the thin section corresponds to epoxy; (**b**) Bulk REE concentrations normalized to standard MuQ values proposed by Kamber, *et al*.^61^; (**c**) δ^30^Si values of quartz crystals measured *in situ* using fsLA-Q-ICP-MS on chert matrix and veins at locations highlighted by arrows in (**a**). The bulk δ^30^Si value measured using MC-ICP-MS on whole rock sample lies in the blue band.

The petrographic thin section studied here was prepared from a hand sample of bedded black chert collected from the ACM at our field site (Fig. 1). The presence of ghosted grains visible as outlines within the microcrystalline matrix suggests the silica has replaced an earlier lithology, at least locally (Fig. 2a). Floating rhombs of ankerite, rhombic voids, and stylolites, suggest carbonate was a significant rock component prior to silicification. Spherical zones of featureless microcrystalline quartz may represent recrystallized impact spherules reported from this unit by Byerly and Lowe^57^, or alternatively, primary silica granules as described by Stefurak, *et al*.^60^. Later generations of hydrothermal veins cross-cut this matrix.

Results of major and minor chemical element concentration analyses on bulk rock sample by ICP-OES, and *in situ* on petrographic thin section by fsLA-Q-ICP-MS, are reported in Tables S1 and S2, respectively. Analytical results of REE element concentrations measured on bulk rock sample using HR-ICP-MS are reported in Table S3. Silicon isotopic compositions and element concentrations measured by MC-ICP-MS on bulk rock sample and in situ using fsLA-Q-ICP-MS are reported in Table S4. Locations of the analyses are indicated by arrows on the photomicrograph of the petrographic thin section (Fig. 2a).

The elemental composition of the whole rock is dominated by SiO_2_ (98 wt%) with minor amounts of Fe, Al, Mg, Ca, Na, Ti, and K (Table S1). Rare Earth Element (REE) concentrations were too low to be quantified *in situ* using the fsLA-Q-ICP-MS method (Table S2). Measurements were thus performed on whole rock samples using HR-ICP-MS. REE concentrations were normalized to standard MuQ values proposed by Kamber, *et al*.^61^. The normalized REE pattern displays LREE depleted compared to HREE and positive La, Eu and Y anomalies (Fig. 2b).

Si isotopic data acquired *in situ* follow the terrestrial mass dependent fractionation line in a three-isotope plot (Fig. S1). δ^30^Si values vary from 0.29 to 0.77‰ (n = 8) in the chert matrix, from 0.40 to 0.52‰ (n = 4) and from −3.69 to −1.39‰ (n = 4) in cross-cutting veins 1 and 2, respectively (Fig. 2c). Considering analytical uncertainties (±0.23‰; 2 SD), quartz crystals in chert matrix and vein 1 have no resolvable internal Si isotopic heterogeneity and have both δ^30^Si values similar to the bulk δ^30^Si value of 0.40 ± 0.11‰ (Fig. 2b). In contrast, the δ^30^Si values of vein 2 are significantly lower and more variable (Fig. 2c). The same contrast is observed in Al/Si mass ratio values measured in the three zones (Fig. 2c), where vein 2 has higher and more variable Al contents than vein 1 and chert matrix.

### Raman microspectroscopy

**Figure 3:**
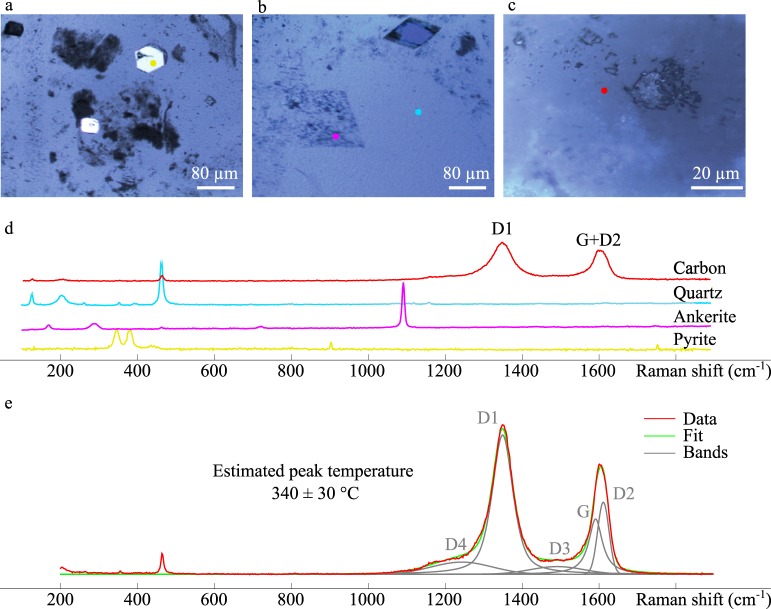
Optical microscopy and Raman spectroscopy analyses. Optical microscope images (**a**–**c**) and associated Raman microspectroscopy data (**d**) showing the presence of cubic/truncated-cubic pyrite (**a**); rhomboedric ankerite (**b**); diffuse carbonaceous matter (**c**) in the chert matrix. Representative Raman spectrum of carbonaceous matter and its decomposition with a pseudo-Voigt function (**e**). Center positions of D1 and D2 bands are 1351.1 ± 1.1 (1 SD) and 1613.1 ± 3.3 (1 SD), respectively. The FWHM parameters of D1 and D2 bands, used for estimating the peak temperature to be 340 ± 30 °C, are 64.3 ± 1.6 (1 SD) and 34.7 ± 2.4 (1 SD), respectively.

Optical microscope images (Fig. 3a–c) and associated Raman microspectroscopy data acquired in the chert matrix (Fig. 3d) indicate the presence of cubic/truncated-cubic pyrite crystals, rhombic ankerite, quartz, and disseminated carbonaceous material. All 10 spectra acquired on carbonaceous matter display similar Raman spectral features characterized by an intense and narrow D1 band and a less intense composite G + D2 band (Fig. 3d,e, red spectra), typical of poorly ordered carbonaceous matter^36,62^. Application of the RSCM (Raman Spectroscopy of Carbonaceous Material) geothermometer^63^ indicates that the maximum burial temperature experienced by carbonaceous matter reached 340 ± 30 °C (Fig. 3e), which is consistent with regional metamorphism in the lower greenschist facies^54^.

### Scanning and transmission electron microscopy

**Figure 4:**
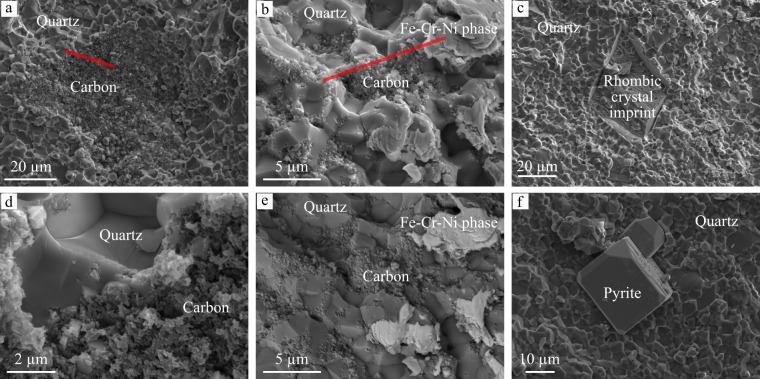
SEM observations of freshly fractured chert fragment surfaces, highlighting carbonaceous matter and the associated mineral phases observed in the silica matrix of the Mount Ada Basalt chert sample (**a**–**d**,**f** are secondary electron images; **e** is a backscattered electron image).

SEM observations of freshly fractured chert fragment surfaces exposing the chert matrix, indicate the presence of disseminated and clustered granular carbonaceous material closely associated with quartz (Fig. 4a,d) and a Fe-Cr-Ni-rich phase (Fig. 4b,e), and the occurrence of rhombic crystals, likely carbonate originally (crystal imprint in Fig. 4c), and cubic pyrite (Fig. 4f) crystals. Red segments indicate where FIB ultrathin foils have been extracted for further characterization using TEM and STXM.

**Figure 5:**
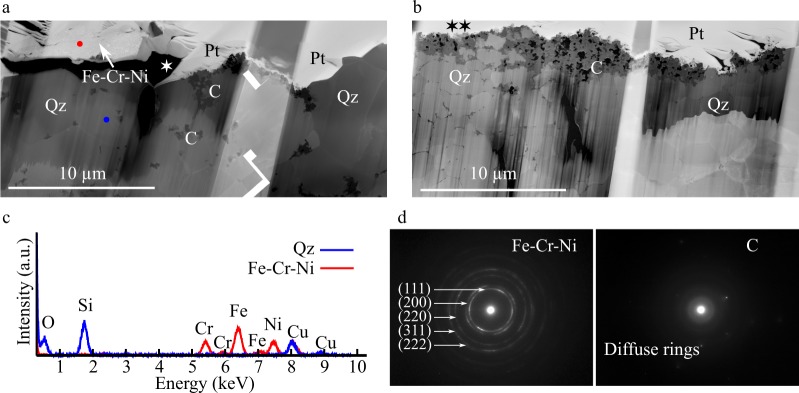
TEM analyses of organo-mineral associations in the extracted FIB foils. STEM images (**a**,**b**) disclose the granular texture of carbonaceous matter. Organic carbon (C), which appears darker than mineral phases, is associated with quartz (Qz) and a Fe-Cr-Ni phase (**a**–**c**), identified as a FCC Fe-Cr-Ni alloy from the electron diffraction pattern displaying concentric rings. (**d**) Whilst organic carbon displays an amorphous structure as indicated by diffuse rings (**d**). The star symbols (⋆; **a**,**b**) indicate where the STXM-based XANES spectroscopy measurements were done (see Fig. 6a). Pt refers to platinum coating used during FIB milling.

TEM images and EDX spectra acquired on the FIB foils highlight the granular texture of carbonaceous matter aggregates (C) associated with quartz (Qz) and the Fe-Cr-Ni-rich mineral phase (Fig. 5a–c). The concentric rings revealed by the electron diffraction pattern corresponding to the Fe-Cr-Ni-rich phase (Fig. 5d) disclose its nano-polycrystalline nature. The crystal spacing of the first five rings from the center spot is measured to be 2.100 Å (1st), 1.816 Å (2nd), 1.280 Å (3rd), 1.091 Å (4th), and 1.044 Å (5th), consistent with a face-centered cubic (FCC) system with (111), (200), (220), (311) and (222) lattice planes, respectively. The mean calculated lattice parameter is 3.627 ± 0.008 Å, which is similar to values reported for FCC Fe-Cr-Ni alloys^64–66^, and significantly lower than values corresponding to Fe-, Cr-, Ni- oxides^67,68^ or sulfides^69,70^ with a cubic unit cell. In contrast, the diffuse rings shown on the electron diffraction pattern corresponding to carbonaceous matter indicate an amorphous structure (Fig. 5d). The few spots visible on this electron diffraction pattern likely correspond to quartz crystals intimately associated with carbonaceous matter.

**Figure 6:**
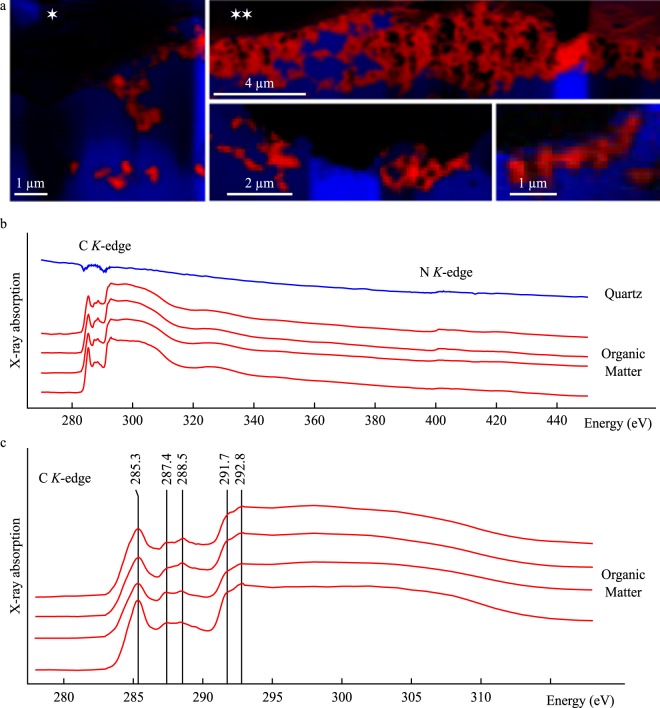
STXM analyses of organo-mineral associations in the extracted FIB foils. STXM maps (**a**) and corresponding X-ray absorption spectra spanning the C- and N- *K* edges (**b**) and C-XANES spectra (**c**) for organic matter (red) and associated quartz (blue). Diagnostic absorption features include: 285.3 eV for aromatic/olefinic groups, 287.4 eV for carbonyl/phenol groups, 288.5 eV for carboxylic groups, 291.7 eV and 292.8 for aromatic groups, The star symbols (⋆) on the STXM maps indicate the corresponding locations on the STEM images of Fig. 5. STXM maps with no star symbols correspond to additional FIB sections investigated using STXM but not TEM.

### STXM-based XANES spectroscopy

STXM maps (Fig. 6a) and corresponding X-ray absorption spectra collected in the 270–450 eV energy range (Fig. 6b) indicate that carbonaceous matter (red) is mostly composed of carbon with no or little nitrogen content while associated quartz (blue) is free of carbon and contains amounts of nitrogen comparable to that of carbonaceous matter (Fig. 6b). Absorption features diagnostic of organic bonds or N_2_ gas are not found at the N *K*-edge for both carbonaceous matter and quartz, indicating that nitrogen is neither organic nor adsorbed atmospheric N_2_.

The C-XANES spectra of carbonaceous matter are similar to one another (Fig. 6c), with an intense peak centered at 285.3 eV, attributed to the presence of 1 s → π* transitions in aromatic and/or olefinic groups^38,71^, two weak peaks centered at 287.4 and 288.5 eV, attributed to 1 s → π* transitions in ketonic/phenolic groups and carboxylic groups, respectively^38,71^, or, less likely, to interlayer states, *i*.*e*. structural defects between aromatic layers described in more structurally ordered carbons^72,73^. The two less intense absorption features, centered at 291.7 eV and 292.8 eV, are attributed to weak or inexistent 1 s → σ* excitonic effects and to 1 s → σ* transitions in aromatic groups, respectively, indicating that the lateral extension of planar domains of highly conjugated aromatic layers, typically observed in carbons experiencing graphitization, did not occur significantly^73,74^.

## Discussion

We investigated the molecular structure and composition of carbonaceous matter, and the mineralogical and geochemical composition of the associated inorganic phases in a chert sample from the 3.47 Ga Mount Ada Basalt Formation. In this section, we discuss the depositional environment and thermal history of these carbon-mineral associations and propose possible origins for carbonaceous matter.

Silicon isotopic and trace element compositions of cherts help constrain the environment in which silica has precipitated (*i*.*e*. hydrothermal vs. marine fluids^75–77^). The near-zero to slightly positive δ^30^Si values (0.29–0.77 ± 0.23‰) of quartz in the chert matrix suggest that silica precipitated in fluids influenced by both seawater and hydrothermal fluids, with negligible terrigenous contribution as indicated by low Al_2_O_3_ content^76–78^. Consistently, the pattern of bulk REE concentrations displays a slightly positive La/La* anomaly (1.3) and a low, yet superchondritic, Y/Ho ratio value (32), both typical of a weak contribution from seawater, whilst the high Eu/Eu* anomaly (2.1) indicates the influence of >250 °C hydrothermal fluids^75,76,79^.

At least two generations of hydrothermal quartz veins crosscut the microquartz chert matrix, one having silicon isotopic and trace element compositions similar to those of the quartz matrix (vein 1), indicating a source similar to the one of quartz in the matrix, the other displaying distinct geochemical characteristics with higher metal content and significantly lower and more variable δ^30^Si (vein 2), possibly indicating the rapid cooling of a distinct hydrothermal fluid^80^.

Together with the δ^30^Si and REE data, the co-occurrence of pyrite and Fe-Cr-Ni alloys in the chert matrix also implies the contribution of geochemically distinct fluids, as Fe-Cr-Ni alloys require very low-sulfur activity, and higher hydrogen fugacity than pyrite, to be stable^81^. Fe, Ni, and Cr are the most abundant transition metals in ultramafic rocks^82^ and their alloys are likely the products of serpentinization reactions, as previously described in the Luobusa ophiolite of Tibet^83^.

Such physicochemical conditions, with the presence of key biochemical elements such as S, Ni, and Fe, are favorable for the development of microbial life^84^. Carbonaceous matter from all cherts studied so far across the Paleoarchean formations of the Pilbara Craton, including the Mount Ada Basalt, displays depleted δ^13^C isotopic values within the range expected for biological fractionation^85^. The relatively widespread occurrence of such ^13^C-depleted organic materials, sometimes associated with distinct morphotypes, in cherts from Paleoarchean hydrothermally influenced environments, has been interpreted by several authors to be the result of biological processes^30,31,86,87^.

Raman data acquired from the Mount Ada Basalt carbonaceous materials indicate a peak metamorphic temperature similar to that experienced by organic microfossils preserved in younger chert from the 3.43 Ga Strelley Pool Formation^7,8,11,13^. Yet, the carbonaceous materials reported here differ significantly from the Strelley Pool microfossils in terms of molecular, morphological, and textural characteristics. The Strelley Pool organic microfossils contain large amounts of heteroatoms constituting various functional groups such as ketones/phenols, imines, nitriles, hydroxyls, carboxyls and amides^13^, and display distinct morphotypes such as films and lenticular forms^8,13^, with a rough surface texture^13^. In contrast, the Mount Ada Basalt carbonaceous matter does not display detectable organic nitrogen, and contains low amounts of organic hydrogen and oxygen organized in ketone/phenol and carboxylic functional groups. Almost exclusively composed of carbon-rich aromatic compounds, they display granular porous textures without any particular morphotype. Such discrepancies in terms of molecular, morphological, and textural characteristics may simply result from the distinct geological processes having affected the transformation of organic matter in the two localities. Organic microfossils from the Strelley Pool cherts were suggested to have been rapidly entrapped in amorphous silica^8,13^, a process that has long been known to finely preserve morphological characteristics of microfossils^39,40,88^, and that was recently experimentally shown to significantly limit the molecular alteration of microbial remains later submitted to temperature conditions ranging from 250 to 300 °C^44,46^. In the case of the organic material studied here, hydrothermal alteration of initially immature biogenic organic matter may have produced aromatic-rich kerogen or pyrobitumen, while destroying the textural and morphological characteristics of the initial biogenic materials. The C-XANES spectra reported here are similar to those of thermally overmatured kerogen and pyrobitumen^71^, which is therefore consistent with hydrothermally altered biological organics. Assuming similar biogenic precursors but distinct geological histories for organic matter in the Mount Ada Basalt and the Strelley Pool cherts, different burial processes could thus account for the observed discrepancies.

Alternatively, the presence of Fe-Cr-Ni alloys in the chert matrix under the strongly reducing conditions that existed in the hydrothermal system could have enabled the abiotic synthesis of CH_4_ and short hydrocarbon chains by the reduction of carbon monoxide and dioxide (CO or CO_2_)^25,27^. Polymerization reactions may subsequently have formed longer hydrocarbon chains^89^ that might eventually have produced the more complex organic materials observed in the Mt. Ada Basalt chert. CO_2_ and bicarbonate and carbonate ions (ΣCO_2_) are not the only single carbon compounds at equilibrium in H_2_-rich hydrothermal fluids^90^. Experimental work demonstrated that single carbon compound speciation in such aqueous systems at temperature and pressure conditions ranging from 150 to 300 °C, and 35 MPa, respectively, is governed by reactions between CO, ΣCO_2_, ΣHCOOH (formic acid HCOOH + formate HCOO^–^), formaldehyde (CH_2_O), methanol (CH_3_OH), and CH_4_^90^. CH_2_O, which could also form from the oxidation of CH_4_^91^, may have subsequently polymerized to form complex insoluble organic matter, as shown by experimental work carried out under alkaline hydrothermal conditions at temperatures ranging from 90 to 250 °C^92,93^. Condensed carbonaceous matter could also have precipitated directly from the reduction of CO_2_ in H_2_-rich serpentinization-derived fluids under temperature conditions ranging from 200 to 300 °C, as suggested by experimental work and thermodynamic calculations^94,95^.

Because of the presence of sulfur - which poisons their formation - native metal alloys were previously believed to be unstable during silica precipitation in the hydrothermal system of the 3.48 Ga Dresser Formation, Pilbara Craton. It has thus been suggested that an abiotic source for CH_4_ and organic matter in these cherts is unlikely^31,86,96^. Contrasting this view, our observations indicate that, despite the the presence of sulfur, native metal alloys were stable in the ACM hydrothermal system, and the reducing conditions could have enabled the abiotic synthesis of organics, as it was previously proposed for organic matter in the iconic 3.46 Ga Apex hydrothermal chert vein^20^. Given the rarity of putative microfossils in Paleoarchean cherts, and the comparatively widespread distribution of amorphous carbonaceous matter, both in stratiform cherts and, in even larger concentrations^29,86^, within chert vein complexes crosscutting the Archean Greenstone Formations^21,29–31,86^, the present results raise questions about the mechanism of formation of much of the organic matter in hydrothermally influenced Archean environments. The molecular composition and geological fate of hydrothermally-generated abiotic organic solids remain poorly constrained^90^. Recent experimental work has shown that initially distinct organic precursors do not evolve the same way during advanced thermal diagenesis in the absence of fluids, resulting in molecularly distinct organic residues after 100 days at 250 °C and 250 bars^38^. This suggests that it may be possible to distinguish different organic precursors from their residues in the geological record. Yet, it remains unclear how distinct organic precursors would evolve in the presence of fluids, and the degree of prior molecular difference that could exist between abiotic and biological sources in such Paleoarchean hydrothermal systems. Thus, much work remains to be done before it will be possible to draw definitive conclusions about the origin of most Paleoarchean organo-mineral associations. Together with experimental investigations of the hydrothermal synthesis and geological alteration of condensed organics, characterization of natural samples will bring further insight into the possible mechanisms of formation of organic matter in Paleoarchean environments. Further documenting the distribution of native metal alloys in other Paleoarchean cherts, such as in hydrothermal chert veins of the 3.48 Ga Dresser Formation that contain CH_4_ of disputed origin^96,97^, as well as in the associated ultramafic rocks that have experienced serpentinization reactions, will be critical to better constrain the processes that have sourced organic matter in volcano-sedimentary environments on the early Earth, and perhaps one day on Mars.

## Material and Methods

### Chert sample

Geological samples were acquired in 2011 and 2018 from Antarctic Creek Member outcrop in the North Pole Dome, Pilbara Region, Western Australia (21° 9.455 S, 119° 19.6050, North Shaw 1:100 000 Map Sheet). Hand samples of black chert were placed in cloth sample bags for transport. Thin sections were made by Wagner Petrographics, UT, USA, following standard techniques. A blue dye was employed to stain epoxy.

### ICP-OES and ICP-MS

#### Whole rock element concentration measurements by ICP-OES

Element concentrations in a pulverized fragment of the chert sample WP0043 were determined after sample digestion using an ICP-OES (Varian 720-ES, HELGES - GFZ, Potsdam, Germany). Analytical methods for sample dissolution and element concentration analyses follow the method described by Schuessler, *et al*.^98^.

Precision and accuracy of the analyses were evaluated through replicate analyses of reference materials processed along with the sample. The results of these measurements and comparison to published results^99–101^ indicate relative uncertainties better than 10% for most elements.

#### Whole rock Si isotope and REE concentration measurements by MC-ICP-MS

The Si isotope composition of the bulk rock sample was determined using a MC-ICP-MS Thermo Neptune (HELGES - GFZ, Potsdam, Germany) after sample dissolution and chromatographic purification of Si following the analytical procedure described in detail previously^102–104^. Repeated sample dissolutions and measurements, and comparison to published data on reference materials (Table S2) indicate that the analytical uncertainties are ± 0.11‰ and ± 0.09‰ (2 SD) for δ^30^Si and δ^29^Si, respectively^104^. ^30^Si/^28^Si and ^29^Si/^28^Si isotopic ratios are reported in the delta notation, relative to the NBS-28 standard value (δ^30^Si/^28^Si_NBS28_ and δ^29^Si/^28^Si_NBS28_, respectively).

REE concentrations were measured using a Thermo Scientific Element HR ICP-MS (HELGES - GFZ, Potsdam, Germany) after sodium peroxide digestion and REE column chromatography separation.

#### In situ Si isotope measurements by fsLA-MC-ICP-MS

The micro-scale Si isotopic composition was determined on a polished petrographic thin section (30 µm thick) of the chert sample using UV femtosecond laser ablation (UV fsLA - GFZ *Fem2*) coupled with a MC-ICP-MS Thermo Neptune (HELGES - GFZ, Potsdam, Germany). Analytical conditions of the UV fsLA-MC-ICP-MS method are briefly described below. Further details are provided by Schuessler and von Blanckenburg^104^.

The UV laser beam (196 nm, <200 fs pulses) was focused beneath the sample surface to obtain a spot diameter of ca. 25 µm at a fluence of ca. 1 J/cm². Ablation was done in raster scanning mode (40 µm/s scan speed) at a laser pulse repetition rate of 60 Hz, ablating a surface area between 100 × 100 µm and 100 × 200 µm with less than 10 µm crater depth. Due to more irregular shapes displayed by quartz crystals in vein 1, line scans were used instead of raster mode.

The chert sample has been analyzed together with reference materials for calibration and analytical quality control (NIST 8546 aka NBS-28, USGS BHVO-2G basalt (glass), IRMM-017 Si single crystal prepared as polished epoxy mounts). Standard bracketing was used for calibration using NBS-28. Each individual Si isotope ratio measurement consists of the mean of 60 1-second integration cycles corrected by on-peak subtraction of the background measured after each sample ablation. Data evaluation followed the protocol described by Schuessler and von Blanckenburg^104^. All time-resolved data were screened to detect potentially occurring irregular mass bias drift, ablation of other phases at depth (not visible on the thin section surface) or any spectral interferences (in a three-isotope-plot). Analysis that did not satisfy with these data acceptance criteria are not reported.

We report results in the delta notation as per mil deviation relative to NBS-28 together with the internal standard error of the mean (2SE) of single sample measurements (Table S1), which is typically <0.2‰ for δ^30^Si, as estimated by error propagation from measurements of one sample and two bracketing standards (NBS-28). The uncertainty of the fsLA-MC-ICP-MS method (external long-term repeatability) is ±0.15‰ and ±0.23‰ (2SD) for δ^29^Si and δ^30^Si, respectively^104^. Verifications of accuracy and precision were done by measurements of reference materials (BHVO-2 and IRMM-017) during this study compared to published data (Table S1).

#### fsLA-Q-ICP-MS element concentration analyses

The chemical composition at the micro-scale was determined by UV femtosecond laser ablation (GFZ Fem2) coupled to a quadrupole ICP-MS (Thermo iCAP-Qc).

Laser ablation was done in raster scanning mode (ca. 25 µm beam diameter, 40 µm/s scan speed, repetition rate 50 Hz, fluence ca. 1 J/cm²) ablating an area of 100 × 100 µm, except for quartz grains in vein 1 of the chert sample, where line scans adapted to the mineral shape were used. Laser ablation locations for element concentrations (fsLA-Q-ICP-MS) were next to the Si isotope analysis locations (fsLA-MC-ICP-MS) in chert matrix and vein 1, i.e. distance of less than 200 µm (except for analyses numbers Z1–05-Si and Z1-05, which are ca. 600 µm apart, and for analyses numbers Z1-10-Si and Z1-10, which are ca. 1600 µm apart. For analyses in vein 2, it was not possible to place Si isotope and concentration analyses locations next to each other, hence, there is no direct spatial correlation between the reported analysis numbers.

The reference material NIST612 (silicate glass) was used for calibration and SRM NIST 616/614/610 were analyzed as unknowns for quality control. Data evaluation was done using the Iolite 3.5 software^105^ and ^29^Si was used as internal standard^106^. For data evaluation of different analysis locations, a constant SiO_2_ concentration of 98 wt% was used as internal standard value (see bulk rock analyses by ICP-OES and in Table S3). Based on ^29^Si signal intensities of all chert laser ablation spots, the micro-scale SiO_2_ relative variability is less than 3%, therefore no bias outside the analytical uncertainties reported with the data is caused by this approach. Accuracy and precision were verified by comparison of our NIST SRM 616/614/610 results to published data^101,107,108^ and measurements of reference materials (IRMM-017 and BHVO-2) during this study (Table S2). The typical uncertainty in fsLA-Q-ICP-MS concentration data is around 10% for most elements (Table S2).

#### Raman spectroscopy

Raman data were obtained using a Horiba Jobin Yvon LabRAM 800 HR spectrometer (MIT, Cambridge, USA) in a confocal configuration equipped with an Ar^+^ laser (532 nm) excitation source and a Peltier Cooled CCD detector. Raman microspectroscopy measurements were performed at constant room temperature, directly on freshly fractured surfaces to characterize the degree of structural organization of carbonaceous matter and to locate the regions of interest for subsequent SEM, FIB, TEM and STXM investigations, and on polished thin section for complementary mineralogical analyses. The laser beam was focused on the sample with a 300 µm confocal hole using a long working distance × 100 objective (NA = 0.8). This configuration provides a ≈1 µm spot size for a laser power delivered at the sample surface below 1 mW, thereby preventing irreversible laser-induced thermal damage^62,109^. A circularly polarized laser using a quarter wavelength plate allows limiting polarization effects. A calibrated edge high band filter (lowest wavenumber: ≈70 cm^−1^) has been used to minimize the elastic backscattered signal^110^. The collected Raman spectra were used to estimate the peak metamorphic temperature experienced by organic matter, based on the work of Beyssac, *et al*.^111^ who first quantified a phenomenological relationship between Raman spectral characteristics and the peak metamorphic temperature. Extraction of spectral parameters from peak fitting procedure and estimation of the peak temperature were done following the methodology proposed by Kouketsu, *et al*.^63^, by using the FWHM-D1 and FWHM-D2 parameters.

#### SEM

SEM was used to locate the carbonaceous materials within the quartz matrix of the chert sample for subsequent extraction using FIB milling. To minimize contamination that may come from sample preparation (polishing, resin), freshly fractured fragments of chert were directly observed after having been analyzed with Raman microspectroscopy. Chert fragments were mounted on aluminum stubs without any additional preparation, except Pt coating. SEM observations were performed on a SEM–field emission gun Supra 55 Zeiss (CNS - Harvard, Cambridge, USA) at a 10-kV accelerating voltage and a working distance of 9 mm.

#### FIB

Four FIB ultrathin foils were extracted from carbon-mineral associations using a FEI Strata DB 235 (IEMN, Lille, France). Milling at low Ga-ion currents allowed minimizing artefacts such as local gallium implantation, mixing of components, creation of vacancies or interstitials, creation of amorphous layers, local composition changes or redeposition of the sputtered material on the sample surface^112,113^.

#### TEM

TEM analyses were performed on FIB foils to document, down to the nanoscale, the morphology and texture of the investigated organo-mineral associations, and to identify the mineral phases. TEM observations were conducted with a JEOL JEM 2010F Field Emission Gun Transmission Electron Microscope (CMSE - MIT, Cambridge, USA) operating at 200 kV. Z-contrast STEM imaging was performed using the high-angle annular dark field mode. High-resolution TEM images were collected using the bright-field mode, which enables to resolve the crystalline planes (of the order of 0.1 nm) of the different mineral phases.

#### XANES spectroscopy

XANES data were collected on FIB sections using the STXM 10ID-1 beamline (SM beamline^114^) at the Canadian Light Source. The 10ID-1 beamline works in the soft X-ray energy range (130–2,500 eV) and is based on an elliptically polarized undulator. The Canadian Light Source storage ring is operated at 2.9 GeV and between 250 and 150 mA current. The microscope chamber was first pumped down to 100 mTorr after sample insertion and back-filled with He gas. A 100-nm-thick titanium filter was used to remove the contribution of second-order light. Energy calibration was done using the well-resolved 3p Rydberg peak of gaseous CO_2_ at 294.96 eV for the C *K*-edge and using the 1 → π* photoabsorption resonance of gaseous N_2_ at 400.8 eV for the N *K*-edge. X-ray absorption spectroscopy was performed by collecting image stacks with a spatial resolution of 25 nm, i.e. by rastering selected areas of the samples in the x–y directions at energy increments of 1 eV over the 270–450 eV energy range using the low-energy grating of the 10ID-1 SM beamline. Additional image stacks were collected at energy increments of 0.1 eV over the carbon (270–340 eV) and the nitrogen (390–450 eV) absorption ranges, to resolve the fine structures near the C and N *K*-edges (XANES spectroscopy). Stack measurements were performed with a dwell time of ≤ 1 ms per pixel to prevent irradiation damage^115^. Stack alignments and extraction of XANES spectra have been done using the aXis2000 software (ver2.1n).

## Supplementary information

Supplementary information

Figure S1

Tables S1-4

## Data Availability

The data that support the findings of this study are available from the corresponding author, J.A., upon reasonable request.
