# Supplementary material for: Organo-mineral associations in chert of the 3.5 Ga Mount Ada Basalt raise questions about the origin of organic matter in Paleoarchean hydrothermally influenced sediments
Source: Sci Rep. 2019 Nov 13;9:16712. doi: 10.1038/s41598-019-53272-5 (PMC6853986; doi:10.1038/s41598-019-53272-5)
Supplement: Supplementary file 1 — Supplementary information [file 41598_2019_53272_MOESM1_ESM.docx]

Supplementary information

Organo-mineral associations in chert of the 3.5 Ga Mount Ada Basalt raise questions about the origin of organic matter in Paleoarchean hydrothermally influenced sediments

Julien Alleon^1†*^, David T. Flannery^2^, Nicola Ferralis^3^, Kenneth H. Williford^2^, Yong Zhang^3^, Jan A. Schuessler^4^, Roger E. Summons^1^

*1 – Department of Earth, Atmospheric and Planetary Sciences, Massachusetts Institute of Technology, Cambridge, Massachusetts, USA.*

*2 – Jet Propulsion Laboratory, California Institute of Technology, Pasadena, California, USA.*

*3 – Department of Materials Science and Engineering, Massachusetts Institute of Technology, Cambridge, Massachusetts, USA.*

*4 – GFZ German Research Centre for Geosciences, Potsdam, Germany.*

^†^ *– Now at Institute of Earth Sciences, University of Lausanne, Lausanne, Switzerland.*

** corresponding author: julien.alleon@gmail.com,*

Supplementary data available online:

Figure S1

*Description*: Three-isotope plot of the silicon isotopic data acquired in situ on the petrographic thin section.

Tables S1-4

*Description*: Geochemical data. Results of major and minor chemical element concentration analyses on bulk rock sample by ICP-OES, and in situ on petrographic thin section by fsLA-Q-ICP-MS, are reported in Tables S1 and S2, respectively. Analytical results of REE element concentrations measured on bulk rock sample using HR-ICP-MS are reported in Table S3. Silicon isotopic compositions and element concentrations measured by MC-ICP-MS on bulk rock sample and in situ using fsLA-Q-ICP-MS are reported in Table S4.
