# Supplementary material for: Organo-mineral associations in chert of the 3.5 Ga Mount Ada Basalt raise questions about the origin of organic matter in Paleoarchean hydrothermally influenced sediments
Source: Sci Rep. 2019 Nov 13;9:16712. doi: 10.1038/s41598-019-53272-5 (PMC6853986; doi:10.1038/s41598-019-53272-5)

Figure S1

Organo-mineral associations in chert of the 3.5 Ga Mount Ada Basalt raise questions about the origin of organic matter in Paleoarchean hydrothermally influenced sediments

Julien Alleon[1†*], David T. Flannery[2], Nicola Ferralis[3], Kenneth H. Williford[2], Yong Zhang[3], Jan A. Schuessler[4], Roger E. Summons[1]

1 – Department of Earth, Atmospheric and Planetary Sciences, Massachusetts Institute of Technology, Cambridge, Massachusetts, USA.
2 – Jet Propulsion Laboratory, California Institute of Technology, Pasadena, California, USA.
3 – Department of Materials Science and Engineering, Massachusetts Institute of Technology, Cambridge, Massachusetts, USA.
4 – GFZ German Research Centre for Geosciences, Potsdam, Germany.
† – Now at Institute of Earth Sciences, University of Lausanne, Lausanne, Switzerland.

** corresponding author: julien.alleon@gmail.com,*

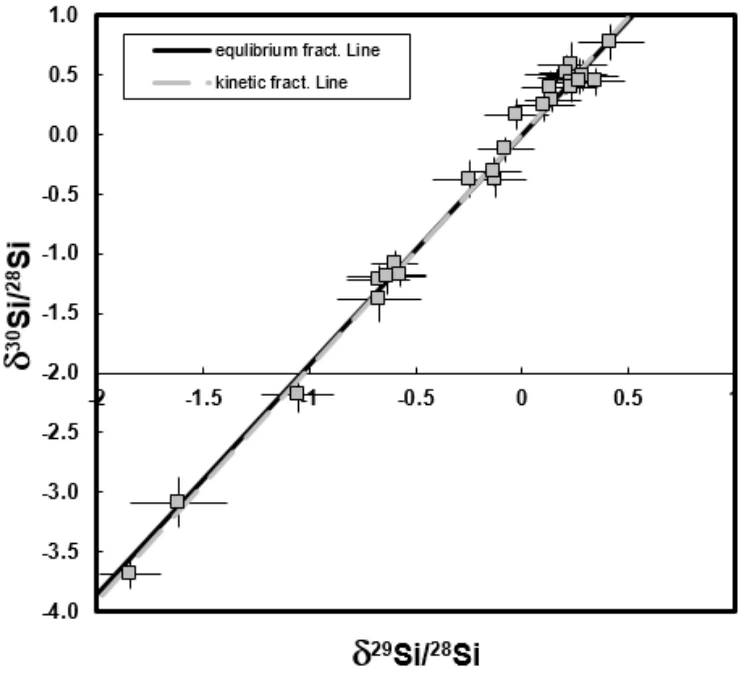

Supplement: Supplementary file 2 — Figure S1 [file 41598_2019_53272_MOESM2_ESM.pdf]
